# Supplementary material for: Mucosal and Serum Neutralization Immune Responses Elicited by COVID-19 mRNA Vaccination in Vaccinated and Breakthrough-Infection Individuals: A Longitudinal Study from Louisville Cohort
Source: Vaccines (Basel). 2025 May 24;13(6):559. doi: 10.3390/vaccines13060559 (PMC12197714; doi:10.3390/vaccines13060559)
Supplement: Supplementary file 1 [file vaccines-13-00559-s001.zip › vaccines-3588306-supplementary.pdf]

Supplementary Materials

Table S1. Mean and standard deviations across different virus types for each age group and comparison between different virus types for each fixed age group.

Variables	Groups	Wuhan	Beta	Delta	Omicron	p_all	p_BW	p_DW	p_OW	p_DB	p_OB	p_OD
**Age Group**	18-40	6554 (7292)	487 (829)	934 (912)	118 (166)	<0.001	<0.001	<0.001	<0.001	<0.001	<0.001	<0.001
	41-60	3856 (6630)	437 (1008)	730 (800)	87 (165)	<0.001	<0.001	<0.001	<0.001	<0.001	<0.001	<0.001
	60 up	3114 (2559)	148 (198)	520 (529)	43 (41)	<0.001	<0.001	<0.001	<0.001	<0.001	<0.001	<0.001

Table S2. Mean and Standard deviations (in parenthesis) across vaccine duration for SARS-CoV-2 VOCs and significant difference amongst each other.

Variables	Groups	Wuhan	Beta	Delta	Omicron	p_all	p_BW	p_DW	p_OW	p_DB	p_OB	p_OD
**Vaccination Duration**	over 6 months	2640 (5942)	147 (339)	466 (510)	56 (78)	<0.001	<0.001	<0.001	<0.001	<0.001	<0.001	<0.001
	under 6 months	6386 (5581)	571 (1003)	994 (906)	110 (180)	<0.001	<0.001	<0.001	<0.001	<0.001	<0.001	<0.001

Table S3. Mean and standard deviations (in parenthesis) across age groups for different VOCs and comparisons between age groups for a particular SARS-CoV-2 VOC.

Type of Virus	18-40	41-60	60 up	p_all	p_18-40 vs 41-60	p_18-40 vs>60	p_41-60 vs >60
**Wuhan**	6554 (7292)	3856 (6630)	3114 (2559)	0.003	0.001	0.034	0.228
**Beta**	487 (829)	437 (1008)	148 (198)	0.001	0.678	0.001	0.003
**Delta**	934 (912)	730 (800)	520 (529)	0.047	0.149	0.014	0.302
**Omicron.BA.1**	118 (166)	87 (165)	43 (41)	<0.001	0.003	<0.001	0.05

Table S4. Mean and standard deviations across virus types for each age group and comparison between different virus types for each fixed age group.

Variables		Wuhan	Beta	Delta	Omicron	p_all	p_BW	p_DW	p_OW	p_DB	p_OB	p_OD
**Age Group**	18-40	15253 (14933)	3642 (3586)	3954 (3561)	1417 (1291)	<0.001	<0.001	<0.001	<0.001	0.497	<0.001	<0.001
	41-60	8208 (9941)	3183 (3378)	3089 (4836)	972 (992)	<0.001	<0.001	<0.001	<0.001	0.241	<0.001	<0.001
	60 up	5134 (5276)	1653 (1549)	2003 (2204)	607 (706)	<0.001	<0.001	<0.001	<0.001	0.193	<0.001	<0.001
**Vaccination Duration**	over 6 months	3002 (3521)	1226 (1788)	1153 (1162)	614 (530)	<0.001	<0.001	<0.001	<0.001	0.77	<0.001	<0.001
	under 6 months	16102 (13010)	4447 (3283)	4892 (4494)	1388 (1315)	<0.001	<0.001	<0.001	<0.001	0.471	<0.001	<0.001

Table S5. Mean and Standard deviations (in parenthesis) across vaccine duration for SARS-CoV-2 VOCs and significant difference amongst each other.

	**>6months**	**<6months**	**p_all**
**Wuhan**	3002 (3521)	16102 (13010)	<0.001
**Beta**	1226 (1788)	4447 (3283)	<0.001
**Delta**	1153 (1162)	4892 (4494)	<0.001
**Omicron.BA.1**	614 (530)	1388 (1315)	<0.001

Table S6. Mean and standard deviations (in parenthesis) across age groups for VOCs and comparisons between age groups for a particular SARS-CoV-2 VOC.

	18-40	41-60	60 up	p_all	p_12	p_13	p_23
**Wuhan**	15253 (14933)	8208 (9941)	5134 (5276)	<0.001	0.022	<0.001	0.059
**Beta**	3642 (3586)	3183 (3378)	1653 (1549)	0.002	0.807	0.001	0.003
**Delta**	3954 (3561)	3089 (4836)	2003 (2204)	0.013	0.319	0.003	0.051
**Omicron.BA.1**	1417 (1291)	972 (992)	607 (706)	<0.001	0.062	<0.001	<0.001

Table S7 and S8. Mean, Standard deviation (in parenthesis) and p value for ID50 values across age groups for Wuhan, BA.1, BA.2 and BA.5 and comparisons between age groups.

Table S7

	Wuhan	Omicron.BA.1	Omicron.BA.2	Omicron.BA.5	p_all	p_BA1W	p_BA2W	p_BA5W	p_BA2_BA1	p_BA5_BA1	p_BA5_BA2
**18-40**	993 (1266)	241 (329)	400 (515)	80 (80)	<0.001	<0.001	<0.001	<0.001	<0.001	<0.001	<0.001
**41-60**	1137 (1695)	194 (211)	372 (243)	134 (97)	<0.001	<0.001	<0.001	<0.001	<0.001	0.856	<0.001
**60 up**	468 (220)	68 (52)	103 (39)	55 (2)	<0.001	<0.001	<0.001	<0.001	0.022	0.92	0.017

Table S8

	18-40	41-60	>60	p_all	p_18-40 vs 41-60	p_18-40 vs >60	p_41-60 vs >60
**Wuhan**	993 (1266)	1137 (1695)	468 (220)	0.821	0.972	0.553	0.548
**Omicron.BA.1**	241 (329)	194 (211)	68 (52)	0.672	0.949	0.381	0.416
**Omicron.BA.2**	400 (515)	372 (243)	103 (39)	0.218	0.933	0.091	0.089
**Omicron.BA.5**	80 (80)	134 (97)	55 (2)	0.139	0.049	0.98	0.225

Table S9 and S10. Mean, Standard deviation (in parenthesis) and p value for IgG endpoint titers across age groups for Wuhan, BA.1, BA.2 and BA.5 and comparisons between age groups.

Table S9

	Wuhan	Omicron.BA.1	Omicron.BA.2	Omicron.BA.5	p_all	p_BA1W	p_BA2W	p_BA5W	p_BA2_ BA1	p_BA5_ BA1	p_BA5_ BA2
**18-40**	5469 (4423)	1146 (879)	1380 (822)	634 (750)	<0.001	<0.001	<0.001	<0.001	0.054	<0.001	<0.001
**41-60**	1684 (1184)	317 (213)	852 (633)	357 (288)	<0.001	<0.001	<0.001	<0.001	<0.001	0.822	<0.001
**60 up**	7249 (3255)	1094 (456)	961 (522)	566 (306)	<0.001	<0.001	<0.001	<0.001	0.195	<0.001	<0.001

Table S10

	18-40	41-60	60 up	p_all	p_18-40 vs 41-60	p_18-40 vs >60	p_41-60 vs >60
**Wuhan**	5469 (4423)	1684 (1184)	7249 (3255)	<0.001	<0.001	0.304	<0.001
**Omicron.BA.1**	1146 (879)	317 (213)	1094 (456)	<0.001	<0.001	0.713	0.003
**Omicron.BA.2**	1380 (822)	852 (633)	961 (522)	0.157	0.048	0.49	0.57
**Omicron.BA.5**	634 (750)	357 (288)	566 (306)	0.368	0.253	0.609	0.224

Table S11. Mean, Standard deviation (in parenthesis) and p value for ID50 values across age groups for Wuhan, BA.1, BA.2 and BA.5 and comparisons between age groups.

	18-40	41-60	60 up	p_all	p_18-40 vs 41-60	p_18-40 vs >60	p_41-60 vs >60
**Wuhan**	1796 (1145)	1666 (1568)	2697 (2040)	0.399	0.37	0.576	0.208
**Omicron.BA.1**	971 (719)	1104 (862)	924 (629)	0.995	0.947	0.927	0.963
**Omicron.BA.2**	1558 (1269)	1566 (1344)	1283 (486)	0.992	0.915	0.915	0.974
**Omicron.BA.5**	1946 (3079)	954 (1310)	1255 (1350)	0.197	0.111	0.873	0.175

Table S12. Mean, Standard deviation (in parenthesis) and p value for IgG endpoint titers across age groups for Wuhan, BA.1, BA.2 and BA.5 and comparisons between age groups.

	18-40	41-60	60 up	p_all	p_18-40 vs 41-60	p_18-40 vs >60	p_41-60 vs >60
**Wuhan**	4430 (2341)	5118 (4496)	5306 (3960)	0.952	0.879	0.854	0.758
**Omicron.BA.1**	2443 (1335)	3331 (3799)	2893 (2144)	0.947	0.83	0.75	0.861
**Omicron.BA.2**	4812 (2404)	5325 (4558)	6230 (3190)	0.743	0.876	0.535	0.442
**Omicron.BA.5**	1917 (1379)	2706 (2427)	2907 (1588)	0.358	0.282	0.18	0.541
